# Socioeconomic Disadvantage, All-Cause and Cause-Specific Mortality in Patients Treated With Maintenance Dialysis: A Mediation Analysis of Geographical Inequity and Multimorbidity

**DOI:** 10.1016/j.xkme.2025.101061

**Published:** 2025-07-02

**Authors:** Farzaneh Boroumand, Wai H. Lim, Shuvo Bakar, Ryan Gately, Pedro Lopez, Dharshana Sabanayagam, Anita van Zwieten, Lin Zhu, Germaine Wong, Armando Teixeira-Pinto

**Affiliations:** 1 Sydney School of Public Health, University of Sydney, Sydney, Australia; 2 School of Mathematical and Physical Sciences, Macquarie University, Sydney, Australia; 3 Center for Kidney Research, Kids Research Institute, The Children’s Hospital at Westmead, Westmead, New South Wales, Australia; 4 School of Medical and Health Sciences, Edith Cowan University, Perth, Western Australia, Australia; 5 Department of Renal Medicine, Sir Charles Gairdner Hospital, Perth, Western Australia, Australia; 6 Medical School, University of Western Australia, Perth, Western Australia, Australia; 7 Department of Kidney and Transplant Services, Princess Alexandra Hospital, Brisbane, Australia; 8 Pleural Medicine Unit, Institute for Respiratory Health, Perth, Western Australia, Australia; 9 Medical School, Faculty of Health & Medical Sciences, University of Western Australia, Perth, Western Australia, Australia; 10 Grupo de Pesquisa em Exercício para Populações Clínicas (GPCLIN), Universidade de Caxias do Sul, Caxias do Sul, Rio Grande do Sul, Brazil; 11 Department of Renal and Transplantation Medicine, Westmead Hospital, New South Wales, Australia

**Keywords:** Area-level socioeconomic status (SES), kidney failure, multimorbidity, dialysis, geographical remoteness, mediation analysis, death

## Abstract

**Rationale & Objective:**

Social gradient in health (a “social gradient in health” refers to the observed pattern in which individuals with lower socioeconomic status typically experience poorer health outcomes than those with higher socioeconomic status. This indicates that health disparities exist across different social levels, with the most disadvantaged groups experiencing the worst health outcomes) is significant and established in patients with kidney failure, but the pathways of this relationship are unknown. We aimed to assess the mediating effects of multimorbidity and geographical remoteness in the socioeconomic status (SES)-death associations.

**Study Design:**

A cohort study.

**Setting & Participants:**

All patients with kidney failure aged 18 years and above, who commenced dialysis in Australia from 2005 to 2019.

**Exposure:**

Area-level SES.

**Outcomes:**

All-cause and cause-specific death.

**Analytical Approach:**

The effect of SES on all-cause and cause-specific death was analyzed using the inverse probability stabilized weighting. Mediating effects of geographical remoteness, diabetes mellitus (DM) and cardiovascular disease (CVD) on the association between lower SES and all-cause and cause-specific death were explored.

**Results:**

A total of 35,239 patients receiving incident dialysis were included, with a median (p25, p75) follow-up period of 3.3 (1.7-5.9) years. Compared with patients from higher SES, the average hazard rate for all-cause death among those from lower SES was 17% higher (total effect [TE] = 0.17, 95% CI [0.12-0.23]). Proportions of the effects between SES and all-cause mortality mediated by geographical remoteness, CVD, and DM were 29.4%, 11.8%, 17.6%, respectively, whereas SES explained 41.2% of the TE directly. Compared with patients from high SES, patients from lower SES have on average a higher hazard rate of CVD (TE = 0.26, 95% CI, [0.15-0.38]) and infection-related deaths (TE = 0.12, 95% CI, [0-0.25]). The effects of SES on CVD and infection-related deaths were mediated by CVD and DM, but not geographical remoteness.

**Limitations:**

Potential residual confounding and other latent mediators.

**Conclusions:**

Geographical remoteness, diabetes, and CVD are potential mediators that lie in the pathways between SES and all-cause and cause-specific deaths. A multifaceted approach with sustained efforts from multiple sectors to address these factors may reduce the social disparities observed in patients treated with dialysis.

## Plain-Language Summary

People with kidney failure who have less money or support often have more health problems than those who are better off. In this study, we found that this is not just because of their financial situation, but also because many also have other health problems, including diabetes and heart disease, and they may live in remote areas where it may be harder to access health care and specialist appointments. Our study suggests that we need to provide more support to these people and make sure everyone with kidney failure has a fair chance at good health, no matter where they live or how much money they have.

Patients with kidney failure undergoing treatment with dialysis experienced a 50-fold increased risk of death compared with an age and sex-matched general population.^1^ There are many factors contributing to the excess risk of death in dialysis populations. These may include the very high vascular disease burden, coupled with a rapid deterioration of co-existing comorbid conditions, such as hypertension and anemia, which are frequently observed after dialysis initiation.^2^ Apart from clinical factors, lower socioeconomic status (SES) is also a significant risk factor for death in patients with kidney failure receiving dialysis treatments.^3^^,^^4^ In a systematic review and meta-analysis of 14 studies, lower SES has been shown to be a risk factor of all-cause and cause-specific death in patients treated with dialysis, and this relationship is consistent across all SES indicators. Patients from lower SES (assessed by income, education attainment, and occupation) were twice as likely to die compared with patients from higher SES backgrounds.^5^ Furthermore, there is evidence to suggest the effects of SES on mortality are modified by factors such as ethnicity and sex/gender. For example, patients of African American descent with lower educational attainment treated with dialysis had higher mortality than White patients after accounting for important clinical and sociodemographic factors.^6^ Socioeconomic disadvantage may also exert its impact on the risk of death among women differently than men treated with dialysis, with women experiencing a high relative risk of infection-related death among those from lower SES compared with male counterparts.^7^

Although many of these modifying effects have been examined in previous literature, previous research has not identified the factors linking lower SES and death on dialysis. Knowledge of these factors are important because modification of these mediators through a concerted and comprehensive approach from multiple health sectors may improve the health outcomes of patients with kidney failure. In addition, there are different pathways through which the various mediators may lie between lower SES and death. The contribution of these mediators is dependent on how some of the earlier mediators affect the later ones. For instance, with the rising costs of living and the housing affordability crisis in urban cities, individuals or families from lower SES may migrate to regional and rural parts of the country. People living in rural and remote parts of Australia may experience reduced access to quality health care, and consequentially, a higher risk of developing chronic illnesses such as diabetes and cardiovascular disease (CVD).^8^^,^^9^ The higher cost of living in urban areas disproportionately affects individuals from lower socioeconomic backgrounds. As a result, people from lower SES groups are more likely to move to regional and remote areas to manage financial pressures. A recent survey found that housing affordability was the third most significant factor influencing internal migration from the city to regional Victoria. In addition, respondents with a diploma qualification were more likely to have relocated from outer Melbourne to rural Victoria, while those with an undergraduate university degree were more likely to settle in a regional city.^10^ In this study, we first evaluated the effects of SES on all-cause and cause-specific deaths in patients treated with dialysis. We then decomposed the total effects of SES on death into the mediated effects of geographical location and comorbid conditions that are highly prevalent in patients with kidney failure, including diabetes and CVD.

## Methods

### Study Population

**Figure 1:**
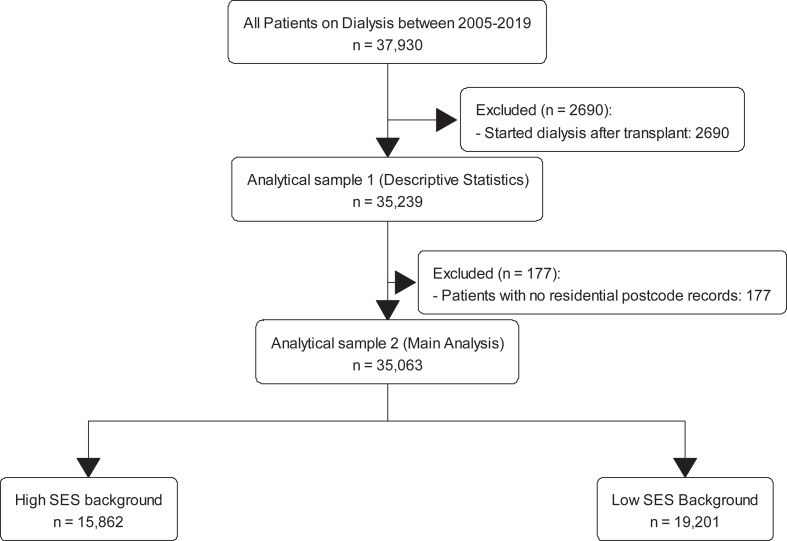
Cohort study flow.

Using data from the Australia and New Zealand Dialysis and Transplant Registry (ANZDATA), all patients with kidney failure aged 18 years or over who commenced peritoneal dialysis or hemodialysis in Australia from 2005 to 2019 were included. Patients with no residential postcode records at commencement of dialysis, recipients who had received pre-emptive kidney transplants, recipients with previous allograft failure, and patients who had recovered from native kidney function within 90 days of dialysis initiation were excluded. This study was approved by the University of Western Australia Human Research Ethics Committee, Perth, Australia (reference number 2021/ET000319). The study flowchart is provided in Fig 1.

### Study Covariates

Baseline characteristics at the time of commencement of dialysis were extracted from ANZDATA, which included age, sex, body mass index, race, dialysis modality (hemodialysis or peritoneal dialysis) at the time of dialysis commencement, prevalent CVD (coronary artery disease, peripheral vascular disease, and cerebrovascular disease), diabetes mellitus, smoking history, primary causes of kidney failure, late referral (defined as referral to a nephrologist < 3 months before dialysis initiation), and dialysis era.

### Exposure Factor

The primary exposure factor was SES derived from residential postcodes recorded at the time of dialysis commencement. The SES was coded into deciles based on the Index of Relative Socioeconomic Advantage and Disadvantage classification by the Australian Bureau of Statistics. The lowest deciles represented the most disadvantaged areas, whereas the highest deciles represented the most advantaged areas. The Index of Relative Socioeconomic Advantage and Disadvantage index is a weighted combination of a census population and housing measures of socioeconomic position, including income, educational level, employment and occupational status, home ownership, and other indicators of relative advantage or disadvantage to classify residential postcodes into percentiles.^11^^,^^12^ It is therefore a global summary of the economic and social conditions of people and households within an area and allows for generalisability and comparisons across regions in the different states and territories in Australia. For this study, we have dichotomized SES into disadvantaged (deciles 1-5) and advantaged areas (deciles 6-10).^12^

### Mediators

**Figure 2:**
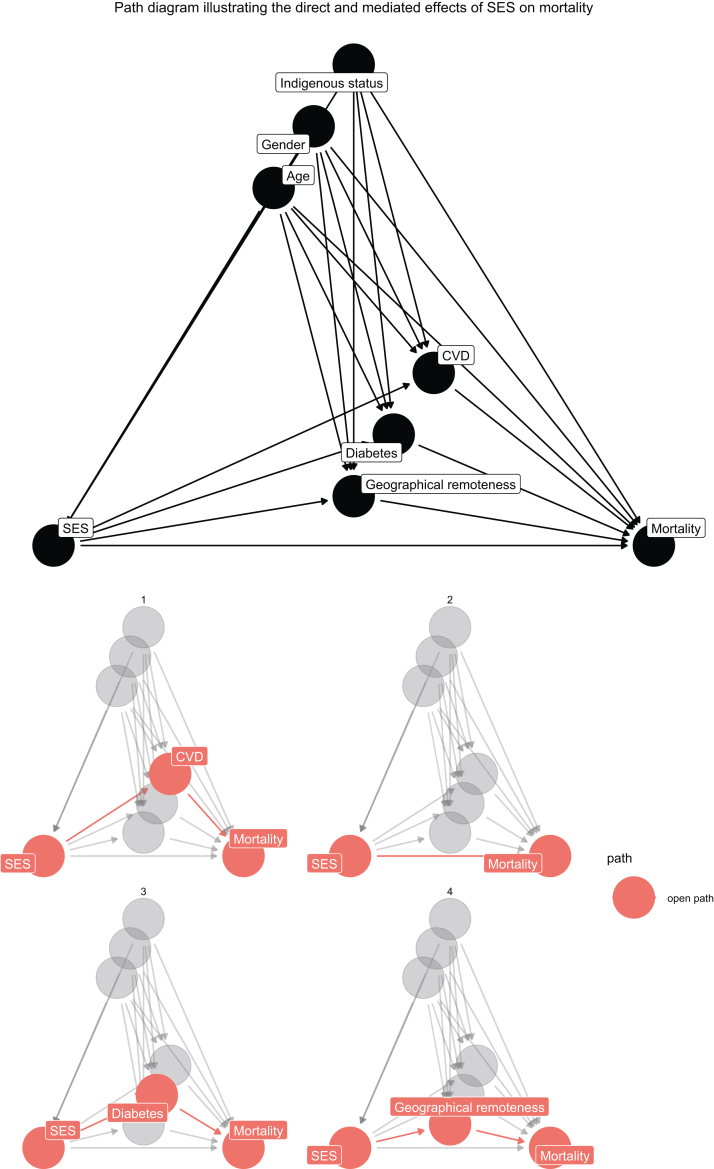
Direct acyclic graph (DAG) showing direct and mediated effects of SES on mortality adjusted for confounders. Top diagram shows DAG of effect of SES on mortality adjusted for confounders. The bottom panel of DAGs illustrating the direct effect of SES on all causes of mortality (2), and mediated effects of SES through geographic remoteness (4), diabetes status (3), and prevalent vascular diseases (1) on all causes of mortality, while adjusting for the confounding effects of age, gender, and Indigenous status.

Three mediators were considered in this study: geographical remoteness, diabetes, and CVD. Residential geographical locations were derived from residential postcodes using the 2006 Australian Standard Geographical classification, and were categorized into major city, regional, and remote regions.^12^ The presence of diabetes and CVD was determined at the time of dialysis commencement (Fig 2).

### Clinical Outcomes

The study outcomes were all-cause mortality on dialysis from CVD, infection, dialysis withdrawal, cancer, and other reasons, and cause-specific death from CVD and infection using predefined definitions provided by ANZDATA (https://www.anzdata.org.au/anzdata/services/data-management/data-forms/). If a patient received a kidney transplant, they would have been censored at the time of transplantation.

### Statistical Analysis

**Figure 3:**
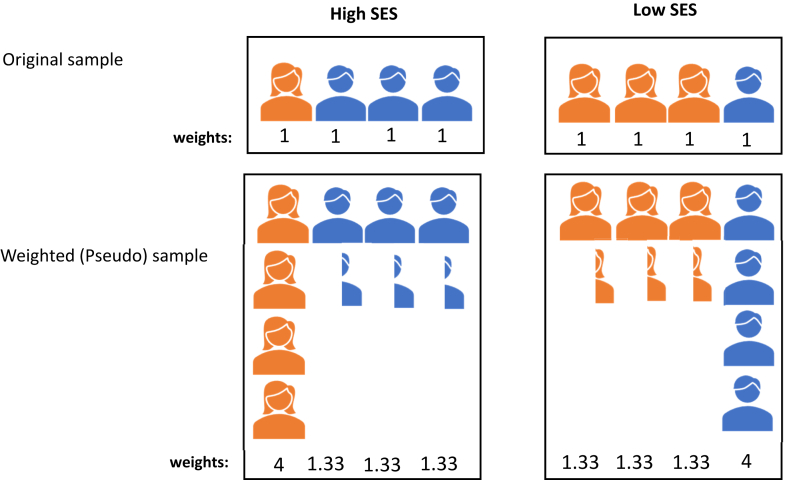
An example of using inverse probability weighting (IPW) to remove the confounding effect of sex. This figure illustrates the benefits of using IPW to remove the confounding effect of sex. In the original sample, the distribution of sex between individuals from high and low socioeconomic status (SES) is different. By applying IPW, each observation in the sample is reweighted to achieve a similar distribution of sex across both SES groups. For instance, in this example, the probability of a male being from a low SES background is 1/4 = 0.25, so the inverse probability weight for males in the low SES group becomes 1/0.25 = 4. Similarly, for females, with a probability of 3/4 = 0.75 of being in low SES, each female in the low SES group receives a weight of 1/0.75 = 1.33. This process is repeated for individuals in the high SES group. Consequently, the total sum of weights for males and females becomes equivalent in both SES groups, whereas in the original sample, this balance did not exist (eg, low SES: 1 male, 3 females and high SES: 3 males, 1 female).

Categorical variables were described using numbers and percentages. For continuous variables, we reported both mean and standard deviation and the median and 25th and 75th quantiles. We conducted a 2-phase data analysis to explore the effects of SES on the time to all-cause and cause-specific death in patients treated with dialysis. In the initial phase, the total effect (TE) of SES on all-cause death was assessed using Inverse Probability stabilised weighting (IPSW) approach. The IPSW approach adjusts for confounding bias by reweighting the observations in a way that the confounders become evenly distributed across the exposure groups.^13^ Fig 3 presents an example showing how IPSW may eliminate confounding effects.

To estimate the IP weights, we applied 2 logistic regression models: one incorporating confounders including age, sex, and indigenous status (Aboriginal and Torres Strait Islander Peoples) and the other excluding them. Subsequently, we calculated the estimated stabilized IP weights using the predicted values from the former model as the denominator and those from the latter model as the numerator. To perform parametric estimation of hazards, we then applied a weighted pooled logistic regression model with a time-varying intercept, enabling the estimation of the temporal change in hazard. This methodology ensures that each person-time observation receives an appropriate IP weight. Therefore, the survival at time “k” was determined by the product of conditional probabilities of having survived each interval between 0 and “k” derived from one minus the hazards.

We then conducted a semiparametric mediation analysis for survival outcome with binary exposure^14^ to assess the effects of SES on all-cause death, mediating through geographical remoteness, diabetes status, and CVD. The mediation analysis was adjusted for the confounders. Fig 2 shows the diagram of the direct and mediated effects of SES on all causes of death. Here, we assessed the total and direct effects of SES on the risk of mortality in patients treated with dialysis. To estimate the uncertainty around these mediation effects, we used the bootstrap method to estimate all effects and their confidence intervals. In addition, we investigated cause-specific mortality, infection, and CVD deaths separately. All statistical significance levels were set at 0.05. The statistical analysis were conducted in R version 4.0.0, for mediation analysis we used the *mma*^15^ package.

## Results

### Baseline Characteristics of the Study Cohort

This study included 35,239 incident patients with kidney failure who initiated dialysis in Australia from 2005 to 2019. Table 1 shows the baseline characteristics of the study cohort stratified by SES. A total of 19,287 (54.7%) patients were from low SES backgrounds. Patients from low SES backgrounds were younger (mean age ± [standard deviation], 61 ± [15] vs 63 ± [16]), most were males (60%), and 15% of patients with low SES were of Aboriginal and/or Torres Strait Islander Peoples. Patients from lower SES backgrounds had higher prevalence of diabetes (54% vs 47%) and CVD (52% vs 50%).

**Table 1:** Baseline Characteristics of the Study Cohort Stratified by Low and High SES

Characteristic	Overall, N = 35,239	Low SES, n = 19,287	High SES, n = 15,952	*P* Value^a^
Age (y), mean ± SD	62 ± 16	61 ± 15	63 ± 16	< 0.001
Age, median (p25, p75)	64 (52-74)	63 (51-72)	66 (53-75)	
Gender, n (%)				<0.001
Male	21,755 (62)	11,657 (60)	10,098 (63)	
Female	13,484 (38)	7,630 (40)	5,854 (37)	
Smoking status,^b^ n (%)				<0.001
Current	4,357 (12)	2,802 (15)	1,555 (10.0)	
Former	14,133 (40)	7,845 (41)	6,288 (39)	
Never	16,328 (46)	8,437 (44)	7,891 (49)	
Unknown	55 (0.2)	27 (0.1)	28 (0.2)	
Indigenous status (Aboriginal and Torres Islander Peoples) n (%)	3,917 (11)	2,960 (15)	957 (6.0)	<0.001
Geographical remoteness^b^^,^^c^ n (%)				<0.001
Major city	23,441 (67)	9,895 (51)	13,546 (85)	
Regional	9,476 (27)	7,656 (40)	1,820 (11)	
Remote	2,322 (6.6)	1,736 (9.0)	586 (3.7)	
Cause of kidney failure, n (%)				<0.001
Diabetic nephropathy	13,422 (38)	7,920 (41)	5,502 (34)	
Glomerulonephritis	6,677 (19)	3,461 (18)	3,216 (20)	
Hypertension	5,216 (15)	2,713 (14)	2,503 (16)	
Not reported	341 (1.0)	174 (0.9)	167 (1.0)	
Other	5,041 (14)	2,557 (13)	2,484 (16)	
Polycystic disease	1,873 (5.3)	947 (4.9)	926 (5.8)	
Reflux nephropathy	704 (2.0)	391 (2.0)	313 (2.0)	
Uncertain	1,965 (5.6)	1,124 (5.8)	841 (5.3)	
Late referral^b^ n (%)				0.002
Late	7,272 (21)	4,099 (21)	3,173 (20)	
Not late	27,652 (78)	15,028 (78)	12,624 (79)	
Kidney transplantation, n (%)	5,682 (16)	2,852 (15)	2,830 (18)	<0.001
Dialysis era, n (%)				0.8
2005-2009	11,393 (32)	6,203 (32)	5,190 (33)	
2010-2014	11,080 (31)	6,077 (32)	5,003 (31)	
2015-2019	12,766 (36)	7,007 (36)	5,759 (36)	
Diabetes^b^^,^^c^ n (%)	17,830 (51)	10,409 (54)	7,421 (47)	<0.001
Chronic lung disease^b^ n (%)	5,847 (17)	3,496 (18)	2,351 (15)	<0.001
Coronary artery disease, n (%)	14,138 (40)	7,915 (41)	6,223 (39)	<0.001
Peripheral vascular, n (%)	8,708 (25)	4,962 (26)	3,746 (23)	<0.001
Cerebrovascular, n (%)	4,997 (14)	2,792 (14)	2,205 (14)	0.091
Prevalent cardiovascular diseases^b^^,^^c^^,^^d^ n (%)	17,975 (51)	10,073 (52)	7,902 (50)	<0.001

a Pearson’s χ ^2^ test; Wilcoxon rank sum test.

b The sum of column percentages may not equal to 100 due to the presence of missing values.

c Mediators.

d Prevalent vascular diseases status is defined based on coronary artery, peripheral vascular, and cerebrovascular.

### Study Outcome Measures

The overall median (p25, p75) follow-up period was 3.3 (1.7-5.9) years, with 3.4 (1.7-5.9), and 3.3 (1.7-5.9) years for low and high SES, respectively. Overall, a total 16,943 (48%) patients died receiving dialysis, whereas 5,682 (16%) received a kidney transplant. Of those who died, the most common causes of death were from CVDs (31%), followed by infections (8.9%), and cancer (4.6%) (Table S1 and Table 1).

### The Effect of SES on All-Cause Mortality

**Figure 4:**
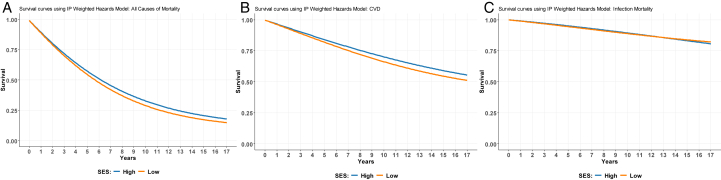
Estimation of the survival curves using IPS weighted hazard model for all causes and cause-specific mortality. (A), survival curves using IPS weighted hazard model for all-cause mortality. (B), survival curves using IPS weighted hazard model for all CVD. (C), survival curves using IPS weighted hazard model for infection mortality. IPS, Inverse Probability stabilised.

The respective one- and 5-year survival probabilities (95% CI) calculated using IPSW for patients from low SES background were 0.888 (0.884-0.892) and 0.544 (0.537-0.551). This compared with the respective survival probabilities of 0.894 (0.889-0.899) and 0.572 (0.564-0.580) for patients from higher SES. Fig 4A shows the estimation of survival curves via IPSW parametric estimation of hazard for all causes of death.

### The Effect of SES on CVD-Related Mortality

The 1-year survival probabilities (95% CI) for individuals from low SES versus high SES were 0.961 (0.959-0.964) versus 0.965 (0.962-0.968), and the 5-year survival probabilities (95% CI) for individuals from low SES versus high SES were 0.819 (0.813-0.824), versus 0.840 (0.834-0.846). Fig 4B demonstrates the estimation of survival curves using IPSW parametric estimation of hazard for CVD-related mortality.

### The Effect of SES on Infection-Related Mortality

The one-year and 5-year survival probabilities (95% CI) for patients from low SES were 0.989 (0.987-0.990) and 0.990 (0.989-0.992), and 0.945 (0.941-0.948) and 0.951 (0.948-0.954) for patients from higher SES. Fig 4C shows the estimation of survival curves using IPSW parametric estimation of hazard for infection-related mortality.

### Mediation Analysis

#### Association Between SES and Mediators

Compared with patients from high SES, the adjusted odd ratios (OR) (95% CI) for diabetes and prevalent CVD among patients from low SES were 1.23 (1.18-1.28) and 1.19 (1.14-1.25), respectively. Compared with patients from higher SES, the odds of living in urban areas among patients from lower SES were 0.22 (0.21-0.23) (Table S2).

#### Association Between Mediators, All-Cause, and Cause-Specific Mortality

Compared with patients living in remote areas, the adjusted HR (95% CI) for death among patients living in urban areas was 0.91 (0.84-0.99). Compared with patients without CVD and diabetes, the adjusted HRs (95% CI) for death among patients with CVD and diabetes were 1.63 (1.58-1.69) and 1.27 (1.23-1.31), respectively. In addition, individuals with prevalent CVD and diabetes exhibited a higher likelihood of CVD-related mortality, with adjusted subhazard ratios (sHRs) of 1.72 (1.62-1.83) and 1.41 (1.33-1.49), respectively. Compared with patients living in remote areas, the sHRs (95%CI) for infection-related mortality among those living in regional areas was 0.75 (0.60-0.94). In addition, individuals with CVD and diabetes exhibited a higher likelihood of infection-related mortality, with the adjusted sHRs of 1.31 (95% CI, 1.17-1.46) and 1.20 (95% CI, 1.08-1.33) (Table S3).

#### Estimation of the Direct, Mediated, and TEs

In this study, we have a single exposure variable, SES, which is binary. We defined the reference category as high SES. Mediation analysis revealed that the TE of SES showed individuals from low SES had a 17% higher probability of death when compared with those from high SES. The estimated hazard rate was 0.17.

Using mediation analysis, we decomposed this TE into 4 components:

1. The direct effect of SES (41.2% of the TE),
2. The mediated effect of SES through remoteness (29.4% of the TE),
3. The mediated effect of SES through CVD (11.8% of the TE), and
4. The mediated effect of SES through diabetes (17.6% of the TE).

These components sum up to 100% of the TE: 41.2% + 29.4% + 11.8% + 17.6% = 100%.

**Figure 5:**
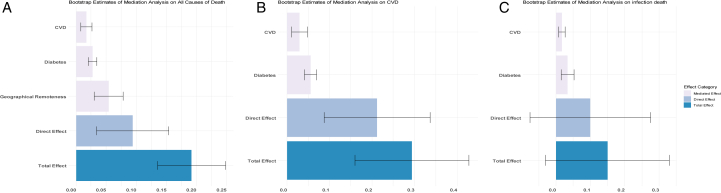
Boxplot of bootstrap estimates of mediation analysis on all causes, CVD, and infection mortality. (A). boxplot of bootstrap estimates of total, direct, and mediated effects of low SES compared to high SES on all causes of mortality for patients treated with dialysis. (B), boxplot of bootstrap estimates of total, direct, and mediated effects of low SES compared with high SES on CVD mortality for patients treated with dialysis. (C), boxplot of bootstrap estimates of total, direct, and mediated effects of low SES compared to high SES on infection mortality for patients treated with dialysis.

Table 2 shows the bootstrap estimates of the direct, mediated, and total effects of SES on all-cause mortality. Compared with patients from higher SES backgrounds, those from lower SES experienced an average higher hazard rate of death (TE = 0.17, 95% CI [0.12-0.23]), indicating those from lower SES experienced a higher probability of death is 17% higher than those from higher SES. The proportions of the effect between SES and all-cause mortality on dialysis mediated by geographical remoteness, CVD, and diabetes were 29.4%, 11.8%, 17.6%, respectively, accounting for 58.8% of the total effect, whereas SES directly explained 41.2% of the total effects. Fig 5A presents a boxplot depicting the bootstrap estimates of total direct and mediated effects.

**Table 2:** Bootstrap Estimates of All Effects of Low SES Compared With High SES on All-Cause Mortality for Patients Receiving Dialysis in the Mediation Analysis

Outcome	Effect	Estimate^a^ (Mean)	SD	95% Bootstrap Confidence Interval	Percentages
All-cause mortality	Total effect of low SES	0.174	0.028	(0.120-0.228)	100%
	Direct effect of low SES	0.081	0.031	(0.021-0.140)	41.2%
	Mediated effect of low SES through geographical remoteness	0.053	0.012	(0.030-0.076)	29.4%
	Mediated effect of low SES through diabetes	0.027	0.003	(0.021-0.032)	17.6%
	Mediated effect of low SES through prevalent CVD	0.015	0.004	(0.006-0.024)	11.8%
CVD mortality	Total effect of low SES	0.260	0.061	(0.149-0.379)	100%
	Direct effect of low SES	0.185	0.059	(0.069-0.301)	70.4%
	Mediated effect of low SES through diabetes	0.049	0.006	(0.037-0.062)	18.8%
	Mediated effect of low SES through prevalent CVD	0.028	0.007	(0.015-0.041)	10.8%
Infection mortality	Total effect of low SES	0.124	0.063	(0.000-0.249)	100%
	Direct effect of low SES	0.096	0.072	(0.045-0.237)	66.1%
	Mediated effect of low SES through diabetes	0.028	0.007	(0.015-0.042)	22.6%
	Mediated effect of low SES through prevalent CVD	0.014	0.005	(0.004-0.023)	11.3%

Abbreviations: CVD, cardiovascular disease; SES. socioeconomic status.

a This value represents the mean of the bootstrap estimates across all replications. For each bootstrap sample, the hazard rate was calculated, and the average of these hazard rates is reported as the bootstrap estimate. Therefore, the sum of the mediated and direct effects may not exactly equal the total effect.

Compared with patients from higher SES, patients from lower SES experienced an average higher hazard rate of CVD-related mortality (TE = 0.26, 95% CI [0.15-0.38]). However, geographical remoteness did not mediate the effects of SES on CVD-related mortality. On the contrary, the effects of SES on CVD-related death were mediated by CVD and diabetes, with CVD and diabetes contributing 10.8% and 18.8%, that is, 29.6% of the TE, whereas the direct effect of SES accounted for 70.4% of the TE (Fig 5B; Table 2).

Patients from low SES experienced on an average a higher hazard rate of infection mortality compared to those from higher SES backgrounds (TE = 0.12, 95% CI [0-0.25]). This effect only was mediated through CVD and diabetes, with CVD and diabetes contributing, 11.3% and 22.6%, that is 33.9% of TE, while the direct effect of SES accounted for 66.1% of the TE (Fig 5C; Table 2).

## Discussion

In this large contemporaneous cohort study of incident dialysis patients spanning 15 years, we found that patients from lower SES experienced a higher risk of all-cause, CVD, and infection-related mortality compared with patients from higher SES. We have, quantified the extent of the factors that mediated the relationship between SES and health outcomes in patients treated with dialysis. We have also shown that over 50% of the total exposure-outcome effect for all-cause mortality was mediated through geographical remoteness, diabetes, and CVD. Remoteness status may explain a substantial proportion of the relationship between SES and all-cause mortality. Meanwhile, CVD and diabetes are key mediators of the relationship between low SES and CVD and infection-related mortality.

Previous research has explored the impact of social disadvantage and health outcomes in patients treated with dialysis.^16^ Globally, lower SES (defined by various individual-level indicators, including income, educational levels, perceived financial status, or neighborhood index) is independently associated with poor overall health and quality of life. Lower SES is also associated with reduced access to care, increased risk of smoking and high alcohol use, and higher risk of household food insecurity and nutritional vulnerability.^17^^,^^18^ People from food insecure households are more likely to consume poorer quality foods, foods that are also high in fat and refined sugar with low nutritional value.^19^ This in turn, may lead to metabolic diseases including hypertension, diabetes mellitus, and obesity, and all of these are risk factors for premature death. In patients suffering from chronic illnesses such as kidney failure requiring dialysis, the impact of SES on their overall health may be even more substantial.^20^ The cumulative impacts of out-of-pockets health expenditure, coupled with altered capacity to engage in employment and the disease burden, which has adversely affected the caregiver’s economic and financial status, may have exacerbated the inequities observed in health outcomes in patients treated with dialysis.^21^

Understanding the reciprocal relationship between SES and health and decomposing the factors that lie in these pathways in patients with kidney failure enables researchers, policy makers, and health professionals to devise novel strategies and interventions to address the social determinants of health and advocate for policies at the local, national, and global levels to promote health equity. We did not adjust for sociodemographic variables and comorbid conditions, as our direct acyclic graph (Fig 2) indicates that these factors act as potential mediators in the relationship between socioeconomic disadvantage and health outcomes. Adjusting for mediators in the analysis could lead to overadjustment bias, potentially underestimating the true effect of socioeconomic disadvantage on health outcomes.^22^ Our findings emphasize the importance of shifting the focus from SES alone to the specific modifiable mediators that link SES to survival in dialysis patients. By identifying remoteness, diabetes, and vascular disease as key factors in this pathway, we highlight actionable targets for reducing disparities in outcomes. Rather than treating SES as a fixed determinant, interventions should directly address these mediators, such as improving health care access in remote areas, enhancing diabetes management, and tackling vascular complications. Acting on these factors has the potential to improve survival and reduce inequities in dialysis care meaningfully. Future research should prioritize evaluating and implementing targeted strategies to mitigate the impact of these mediators. In this study, we found that geographical remoteness is a major driver for disparate health outcomes among those from disadvantaged backgrounds. This is not unexpected, as access to care in rural and remote areas is limited by many factors, including fewer specialist care, lack of modern health care technologies, limited dialysis infrastructure, reduced access to transplantation services, and higher costs of delivering care.^23^ Delayed chronic kidney disease diagnosis and late referral are also common for patients living in rural and remote areas. Late referral has been shown to be associated with higher rates of complications, comorbid conditions, and death.^24^ Once diagnosed with kidney failure, patients are required to travel long distances to receive the dialysis training and treatments. The lack of specialist care in rural and remote areas may pose many challenges when patients with complex illness seeking care and include, delay in appropriate diagnostic testing and treatments and lack of access to relevant medical and surgical expertise and these barriers may also be amplified by other social factors such as low health literacy, cultural differences, and poverty, and medical factors such as multicomorbid conditions. Addressing these downstream factors by increasing diagnostic services and different models of care such as developing the necessary infrastructure for e-Health and mobile dialysis units, may transform care delivery for rural and remote communities and enables patients with complex needs to attend medical appointments closer to home. Our study findings also suggested that the presence of diabetes and vascular disease are potential intervening factors that may bridge the health gaps between social disadvantage and infection and CVD-related mortality. In Australia, universal health coverage via the Medicare ensures all residents have free health care services irrespective of their insurance or financial status. Despite the provision of free health care, rural and remote households experience increased financial pressure due to extremely high out-of-pocket costs in accessing chronic kidney disease treatment and other health related care.^23^ Improved access to specialist services through telemedicine and modern technology may enhance accessibility and efficiency, allow remote review and assessments of glucose monitoring, and promote active shared decision-making between patients and their health care professionals. In addition, virtual and in-person patient navigators may improve the care coordination and assist vulnerable patients to seek medical care, and to overcome the many barriers (language, cultural, and financial) that patients from low SES backgrounds may experience.^25^

Removing confounding bias from observational studies poses significant challenges, necessitating the use of specific statistical methodologies. One of these challenges involves addressing the confounding effects. Although randomization can effectively eliminate confounding bias in interventional studies, this option is not viable in observational research. Consequently, we must apply methodologies capable of mitigating confounding effects. Merely including confounders in the model does not guarantee elimination of their influences.

In this article, we implemented inverse probability stabilized weights as a method to address confounding effects by reweighting observations. This approach aims to achieve a balanced distribution of confounders between low SES and high SES backgrounds, resembling the effect of randomization. Specifically, we applied this technique to analyze time-to-event data, seeking to estimate the overall impact of SES on mortality. Additionally, we conducted a mediation analysis to assess how much of this TE could be mediated through factors such as geographical remoteness, prevalent CVD, and diabetes. Using this framework, we could address the complex relationships using time-to-event analysis in the presence of competing events.

Our study effectively uses complex statistical methodologies to address confounding bias when examining associative relationships using observational data. However, our study has some limitations. Despite our best efforts, we were unable to account for other unobserved confounders in the analyses, as some of these factors, such as hospitalisation and metrics for access to care were not being captured within the registry. In addition, the cumulative impact of these unmeasured factors including drug doses, blood pressure, blood sugar levels, and other biochemical parameters, may have an ordered mediated effect on mortality and may be more appropriately assessed using sequential mediation analyses. Given the lack of individual patient SES measures within the registry, area-level SES measures were used as proxies. As such, there is a chance of exposure misclassification, and may result in attenuation of the SES effects on mortality.^26^ This limitation arises because area-level SES may not accurately reflect the specific socioeconomic conditions of individuals within the same region, leading to potential misinterpretations when assessing the association between SES and individual health outcomes. Furthermore, area-level SES measures do not account for key individual-level determinants such as education, income, employment, and financial status, all of which can significantly impact health outcomes. While area SES may influence health care access, it does not fully capture personal lifestyle factors or behaviors, which are often shaped by an individual’s unique economic and educational circumstances. Additionally, there is substantial heterogeneity within areas where some individuals in lower SES regions may have higher incomes or skilled occupations. In comparison, higher SES regions may still include households with lower incomes or less-skilled jobs. These factors complicate the interpretation of SES effects based solely on area-level measures.^27^ Although the literature includes more granular and diverse types of area-level data, such as large-scale data like street-level images, which help better understand neighborhood inequalities and the influence of the built environment on disease outcomes, such detailed data are not provided by the ANZDATA registry.^28^ In addition, the Index of Relative Socioeconomic Advantage and Disadvantage scores were derived from census data and therefore, we were unable to capture the individual-level variations. potential cultural differences that may have contributed to socioeconomic disadvantages and the temporal changes of an individual patient when their socioeconomic conditions altered over time. Potential misclassification of the mediator may also occur as remoteness also relies on aggregated data derived from post codes. For example, patients living in remote areas may still have access to quality care and resources as infrastructure and facilities may change over time. Finally, we have not explored the bidirectional relationship between SES and remoteness on death. As rising costs of living and housing prices emerged from a growing trend of urban gentrification, it may cause displacement of the vulnerable (patients from lower SES) to regional and remote areas of Australia and thus increase health disparities across socioeconomic status. Although the association between SES and remoteness is incontestable, studies have shown that remoteness can further exacerbate SES inequities through reduced access to quality care, relative social isolation, and poor health literacy and subsequently lead to poor health outcomes.

In conclusion, we have shown, for the first time, the complex and yet interrelated relationships between socioeconomic disadvantage and death in patients treated with dialysis are mediated by not just a single factor, but the joint effects of multicomorbid conditions and remoteness. Multimodel interventions that target these factors may reduce the socioeconomic disparity in survival and help to improve the social fairness and sustainability in patients with kidney failure.
