# Supplementary material for: Socioeconomic Disadvantage, All-Cause and Cause-Specific Mortality in Patients Treated With Maintenance Dialysis: A Mediation Analysis of Geographical Inequity and Multimorbidity
Source: Kidney Med. 2025 Jul 2;7(9):101061. doi: 10.1016/j.xkme.2025.101061 (PMC12362684; doi:10.1016/j.xkme.2025.101061)
Supplement: Supplementary File (PDF) — Tables S1-S3. [file mmc1.pdf]

Table S1: Distribution of the number of deaths on dialysis with the specific causes and time to death (days and year) by SES.

Characteristic	Overall, N = 35,239	Low SES, N = 19,287	High SES, N = 15,952	p-value[1]
**Death on Dialysis, n (%)**	16,943 (48)	9,423 (49)	7,520 (47)	0.001
**Causes of Death, n (%): Cancer**	778 (4.6)	401 (4.3)	377 (5.0)	<0.001
**Cardiovascular**	5,333 (31)	3,065 (33)	2,268 (30)	
**Infection**	1,508 (8.9)	864 (9.2)	644 (8.6)	
**Not reported**	172 (1.0)	90 (1.0)	82 (1.1)	
**Other**	3,409 (20)	1,925 (20)	1,484 (20)	
**Withdrawal**	5,743 (34)	3,078 (33)	2,665 (35)	
**Time to Death on Dialysis (Year), Mean (SD)**	4.2 (3.2)	4.2 (3.2)	4.1 (3.2)	0.2
**Time to Death on Dialysis (Year), Median (p25, p75)**	3.3 (1.7, 5.9)	3.4 (1.7, 5.9)	3.3 (1.7, 5.9)	

[1] Wilcoxon rank sum test; Pearson's Chi-squared test

Table S2: Association between exposure (SES) and mediators (Geographical remoteness, diabetes, and prevalent CVD) for patients on dialysis adjusted for confounders

Factors	levels	OR[1] (outcome: diabetes)	OR[1] (outcome: prevalent CVD)	OR[2] (outcome: Geographical remoteness[3])
**SES**	High			
	Low	1.23 (1.18-1.28, p<.001)	1.19 (1.14-1.25, p<.001)	0.22 (0.21-0.23, p<.001)
**Age (every 10 years increase)**		1.20 (1.19- 1.22, p<.001)	1.58 (1.55- 1.60, p<.001)	1.00 (0.98- 1.01, p=.633)
**Gender**	Male			
	Female	0.93 (0.89-0.97, p=.002)	0.67 (0.64-0.70, p<.001)	0.98 (0.93- 1.03, p=.580)
**Indigenous status**	Other			
	Aboriginal and Torres Strait Islander	5.74 (5.26-6.25, p<.001)	1.80 (1.67-1.93, p<.001)	0.03 (0.03- 0.03, p<.001)

[1] Multivariable binary logistic regression
[2] Multivariable ordinal logistic regression
[3] Intercepts (std) for the multivariable ordinal logistic regression: remote areas/reginal areas -3.38 (0.063), and reginal areas/major cities -0.46 (0.054))

Table S3: Association between mediators and all-cause and cause-specific mortality for patients on dialysis adjusted for confounders

Factors	levels	HR (multivariable)	SHR (CR CVD mortality multivariable)	SHR (CR infection mortality multivariable)
**Geographical remoteness** *	Remote			
	Regional	1.01 (0.93-1.09, p=.846)	1.03 (0.89- 1.18, p= 0.71)	0.75 (0.60- 0.94, p=0.013)
	Major cites	0.91 (0.84-0.99, p=.024)	1.05 (0.91- 1.21, p= 0.51)	0.81 (0.64- 1.01, p=0.058)
**Indigenous status**	Aboriginal and Torres Strait Islander	1.14 (1.06-1.22, p<.001)	1.14 (1.02- 1.27, p= 0.021)	1.28 (1.06- 1.55, p= 0.010)
**Age (every 10 years increase)**	Mean ± SD	1.47 (1.45- 1.49, p<.001)	1.09 (1.07- 1.12, p<0.001)	1.14 (1.10- 1.18, p<0.001)
**Gender**	Male			
	Female	1.03 (1.00-1.06, p=.078)	0.93 (0.88- 0.99, p=0.017)	1.16 (1.05- 1.29, p=0.003)
**Diabetes** *	No			
	Yes	1.27 (1.23-1.31, p<.001)	1.41 (1.33- 1.49, p<0.001)	1.20 (1.08- 1.33, p<0.001)
**Prevalent vascular diseases** *	No			
	Yes	1.63 (1.58-1.69, p<.001)	1.72 (1.62- 1.83, p <0.001)	1.31 (1.17- 1.46, p<0.001)
**n=35063, events=16900, Likelihood ratio test=6330.96 on 7 df (p<.001)**<br>*** Mediators**				
